# COVID-19 hospital indoor environments and how it helped patients’ recovery and staff’s work: a case study in South Korea

**DOI:** 10.3389/fpsyg.2023.1192842

**Published:** 2023-07-06

**Authors:** Sang Hee Park, Hye-Kyung Shin, Kyoung-Woo Kim

**Affiliations:** Department of Building Research, Korea Institute of Civil Engineering and Building Technology, Goyang-si, Republic of Korea

**Keywords:** COVID-19 hospitals, occupant perception, patients and staff, perceived helpfulness, indoor environment, acoustic environment

## Abstract

The indoor environment has been recognized as a crucial factor that can influence health and wellbeing of occupants. This is particularly true in hospital settings, where various environmental attributes can significantly affect patients’ recovery and staff members’ productivity. The present study aimed to investigate how occupants in hospitals perceived indoor environment, focusing specifically on COVID-19 hospitals across Republic of Korea. The study recruited two groups of participants: patients (*n* = 100) who had been hospitalized in COVID-19 hospitals and staff members (*n* = 103) who worked in COVID-19 hospitals. The data collected from the participants were analyzed using multiple regression models to determine which environmental attributes significantly affected their perception of the indoor environment. The study revealed that satisfaction with indoor acoustic environment and odor were significant predictors for how patients perceived the indoor environment as helpful for their recovery from COVID-19. On the other hand, odor was also the significant factor affecting staff members’ perceived helpfulness for work. The results suggested that different environmental attributes can have a significant impact on the perception of the indoor environment, depending on the characteristics of occupancy. The study’s findings provided insights into the certain environmental factors that COVID-19 hospitals can prioritize. These insights can help policymakers and hospital administrators to develop strategies to create hospital environments that meet the needs of both groups. The study also suggested that further research is needed to investigate additional factors affecting occupants’ perception of the indoor environment in hospital settings.

## 1. Introduction

An optimal acoustic environment in healthcare facilities is important to promote patients’ health. It is also vital to facilitate effective communication among healthcare professionals. However, hospitals are frequently associated with a noisy setting, where various noise events are caused by medical equipment and alarms, staff conversations, and foot traffic (Basner et al., 2014; Jerlehag et al., 2018; Lam et al., 2022). Despite recommendations from the World Health Organization (WHO) that noise levels in hospital ward should not exceed 35 dB during the day and 30 dB at night (Berglund et al., 1999), empirical evidence has shown that noise levels in hospitals often surpass these guidelines. For instance, a study conducted in the United States measured sound pressure levels in operating rooms and observed that the highest L_*eq*_ ranged from 62 to 66 dBA, depending on the nature of the surgery (Kracht et al., 2007). Moreover, peak levels exceeding 100 dB accounted for over 40% of the total time period. Another study conducted in the United Kingdom examined the acoustic environment in a geriatric ward (Jerlehag et al., 2018). Although the number of beds in a room influenced indoor noise levels, the study reported L_*eq*_ ranging between 50 and 60 dBA were commonly observed during the daytime and evening in 4-bed rooms.

The detrimental effects of noise pollution in hospital settings have been recognized in the literature (Awada et al., 2021). Indoor hospital noise associates with patients’ sleep disruption and has negative physiological impacts on patients, including altered brain activity and impaired cardiovascular function, which may ultimately contribute to patient morbidity (Parthasarathy and Tobin, 2004; Xie et al., 2009; Buxton et al., 2012). In order to find measures to reduce the adverse effects, a study conducted in a Swedish hospital investigated the effects of implementing good and poor acoustic environments by altering the ceiling tiles on patients’ physiological states (Hagerman et al., 2005). The results of the study revealed a significant decrease in pulse amplitude during nighttime in the acute myocardial infarction and unstable angina pectoris groups when the good acoustic setting was implemented, with a higher rate of rehospitalization observed for those treated in the poor acoustic setting. Additionally, patients in the good acoustic environment rated staff attitude more positively than those in the poor acoustic setting. Not only can hospital noise affect physiological states, but it can also have a significant impact on patients’ psychological states. A study investigating the relationship between hospital noise and patients’ wellbeing, conducted through a survey in a Portuguese hospital, found that patients’ subjective wellbeing had a significant relationship with noise events caused by medical equipment and environmental factors (Cunha and Silva, 2015).

The hospital environment plays a crucial role in promoting health and wellbeing of its occupants. Despite the acknowledged significance of the acoustic environment, other environmental factors such as lighting, air quality, temperature, and spatial design, also have impacts on overall quality of the hospital environment. A study conducted in Greece assessed the indoor environmental quality (IEQ) of operating rooms in nine hospitals (Dascalaki et al., 2009). The study surveyed staff to evaluate their perception of IEQ and examined acoustic comfort by measuring perceived noise levels, noise-related symptoms, and potential sources of noise. The results indicated that poor space layout resulted in high noise levels and low satisfaction with the acoustic environment. Another study examined the IEQ of inpatient and outpatient areas in two Chinese hospitals (Liu et al., 2018). The study evaluated whether different IEQ properties met existing standards and analyzed occupants’ satisfaction with the IEQ. The results revealed that indoor air quality was the least satisfactory attribute, and the measured environmental conditions were not highly correlated with subjective satisfaction. A study conducted in Spain analyzed short-term impacts of chemical air pollution, traffic noise, and thermal extremes on emergency hospital admissions due to anxiety, dementia, and suicides in Madrid (Díaz et al., 2020). The study reported that none of the considered chemical pollutants associated with the dependent variables. However, values of the L_*eq*_,_*day*_ were found to be associated with anxiety, depression, and suicides. Additionally, a significant relationship was found between temperature and admissions for anxiety. Although the study’s focus was not directly related to hospital noise, it is essential to consider these findings in the holistic approach of assessing the overall environmental factors that influence occupants’ health and wellbeing. As of now, there is limited understanding of the effects of acoustic factors on occupants’ perception and the relationships between different attributes of IEQ. Additionally, it is imperative to conduct further research to investigate the influence of each attribute of hospital indoor environments on their occupants.

Following the outbreak of COVID-19, hospitals across the globe have experienced a significant increase in patient admissions. One of the critical issues arising from this situation is the shortage of beds, medical equipment, and healthcare professionals. The impact of these challenges on the quality of safe patient care and occupational performance has been recognized (Winkelmann et al., 2022; Safdari et al., 2023; Stayt et al., 2023). Studies have emphasized the importance of giving additional attention to the indoor environment of healthcare facilities. This includes implementing decontamination strategies for indoor air, due to the heightened risks that come with healthcare crises (Elsaid and Ahmed, 2021; Piscitelli et al., 2022). In early 2022, Republic of Korea recorded the highest number of confirmed cases, with 621,127 cases reported on March 17th of the same year. The country also suffered from a lack of resources and the resulting aftereffects, leading to poor quality of patient care.

Indoor environmental quality has been recognized to have a significant impact on occupants’ health and wellbeing, both physiologically and psychologically. It is therefore important to study the effects of IEQ, especially during the COVID-19 pandemic period, when maintaining good indoor environments is more crucial than ever. However, there is a lack of research conducted during this period. This might be attributed to the challenges faced in accessing healthcare facilities. Therefore, the objective of the present study was to examine the perceptions of occupants who had experienced using COVID-19 healthcare facilities. The study aimed to address the following research questions:

(1-1) How do patients’ perceptions of the indoor environment in COVID-19 hospitals affect their recovery from COVID-19?
(1-2) How do staff’s perceptions of the indoor environment in COVID-19 hospitals affect their work at the COVID-19 ward?
(2) How does noise annoyance impact individuals’ perceptions of the acoustic environment in COVID-19 hospitals? (Applicable to both patient and staff groups).

## 2. Materials and methods

### 2.1. Participants

#### 2.1.1. Patient group

The study recruited individuals who were 19 years or older and had experienced hospitalization due to COVID-19. In total, 106 responses were collected from the patient group, and after excluding six outliers, 100 responses were included in the final analysis (Table 1). The outliers were removed because they reported a date of hospitalization later than the survey date. The sample consisted of 51 males and 49 females, with ages ranging from 25 to 78 years (mean = 45.6, SD = 12.1). The majority of respondents reported being hospitalized in 2022, with the length of hospitalization ranging from one to thirty days (mean = 9.7, SD = 5.3) and the number of patients per ward ranging from one to ten people (mean = 3.4, SD = 2.2). The participants were asked to provide the name of the city and province where the hospital they were admitted to was located. The results revealed that the participants were admitted to hospitals located in 74 different provinces.

**TABLE 1:** Demographic characteristics of the survey respondents (*N* = 203).

Demographic characteristics		Patient group	Staff group
		*n* = *100*	*n* = *103*
Sex	Male	51	45
	Female	49	58
Age	20 s	5	15
	30 s	35	34
	40 s	24	30
	50 s	19	20
	60 s and older	17	4
Year of hospitalization	2020	4	–
	2021	37	–
	2022	59	–
Time length of hospitalization	7 days or less	48	–
	More than 7 days	52	–
Total number of patients in the same ward	1 (single-bed)	19	–
	2 to 3 people	36	–
	4 to 5 people	26	–
	More than 6 people	19	–

**FIGURE 1:**
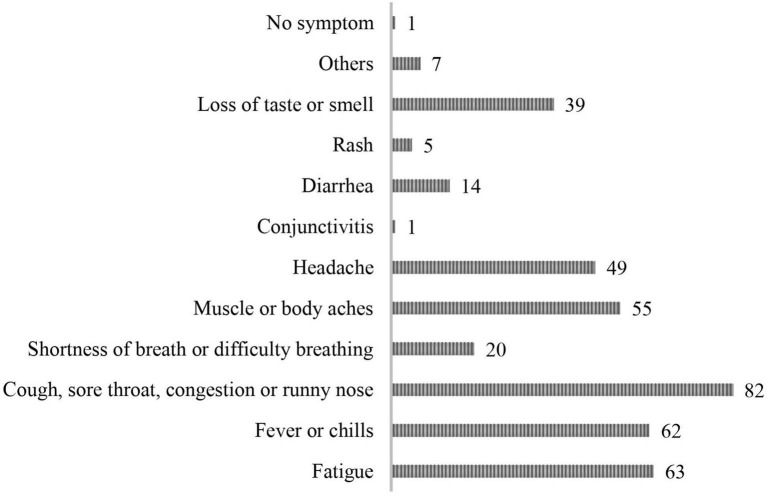
Major symptoms of COVID-19 that the patient group experienced (multi-selection allowed).

Figure 1 displays the major symptoms reported by the respondents, with the question allowing for multi-select options. The largest number of respondents (*n* = 82) experienced cough, sore throat, congestion, or runny nose. The second highest percentage of symptoms reported were fatigue (*n* = 63) and fever or chills (*n* = 62).

#### 2.1.2. Staff group

The study recruited medical staff who were 19 years or older and worked at the COVID-19 hospitals at the time of survey. A total of 110 respondents participated in the study, and seven outliers were removed from the data analysis. Outliers were defined as individuals who reported working at the hospital for longer than 3 years. Since the first confirmed case in Republic of Korea occurred on 20th January 2020, data from individuals who reported a period longer than 3 years were excluded. As shown in Table 1, final 103 responses were used for the data analysis (35 doctors and 68 nurses). The participants consisted of 45 males and 58 females, with ages ranging from 23 to 68 years (mean = 40.6, SD = 10.4). The majority of respondents (*n* = 98) reported they had past experience of working in other ward before joining the COVID-19 ward. The staff were employed in hospitals located in 75 different provinces.

### 2.2. Questionnaire

Table 2 presents question items utilized in the survey to assess the respondents’ perception of the indoor environment. First, the survey measured three items pertaining to satisfaction with acoustic environment. Two were used to assess perceived satisfaction with indoor and outdoor acoustic environments, respectively, based on previous research (Tang et al., 2020). Additionally, another was on speech privacy for this factor is known to be significant for both patients and medical staff in hospital settings (Piscitelli et al., 2022; Winkelmann et al., 2022). Second, satisfaction with visual environment was examined. Studies have shown that both natural and artificial lightings have impacts on hospital occupants’ perception (Tang et al., 2020; Elsaid and Ahmed, 2021). The present study employed two question items that asked the respondents about their satisfaction with natural and artificial lightings. Moreover, since visual privacy is a crucial factor affecting occupants’ perception (R Core Team, 2021; The jamovi project, 2022), the survey included a question on satisfaction with visual privacy. Third, the survey used question items to measure the respondents’ satisfaction with thermal environment (Liu et al., 2018; Fox and Weisberg, 2020), including questions about indoor temperature and humidity. Finally, satisfaction with air quality (Seden et al., 2021) was assessed using question items on odor and air quality. Cronbach’s Alpha (α) values were derived to assess the internal consistency of the question items and are shown in Table 2.

**TABLE 2:** Question items used in the survey and their reliability checked with Cronbach’s Alpha (α).

Variables		Patient group		Staff group	
		Question item	α	Question item	α
Satisfaction with indoor environments 5-point scale (1 “Not satisfied at all” ∼ 5 “Totally satisfied”)	Acoustic environment	Were you satisfied with the indoor acoustic environment?	0.780	Are you satisfied with the indoor acoustic environment?	0.761
		Were you satisfied with the outdoor acoustic environment heard from indoors?		Are you satisfied with the outdoor acoustic environment heard from indoors?	
		Were you satisfied with the speech privacy?		Are you satisfied with the speech privacy?	
	Visual environment	Were you satisfied with the natural lighting?	0.829	Are you satisfied with the natural lighting?	0.791
		Were you satisfied with the artificial lighting?		Are you satisfied with the artificial lighting?	
		Were you satisfied with the visual privacy?		Are you satisfied with the visual privacy?	
	Thermal environment	Were you satisfied with the temperature?	0.887	Are you satisfied with the temperature?	0.862
		Were you satisfied with the humidity?		Are you satisfied with the humidity?	
	Air quality	Were you satisfied with the odor?	0.847	Are you satisfied with the odor?	0.841
		Were you satisfied with the other air quality?		Are you satisfied with the other air quality?	
Annoyance with different noise events 5-point scale (1 “Not annoyed at all” ∼ 5 “Completely annoyed”)	Same space	Were you annoyed with the voice of others made in your ward?	0.888	Are you annoyed with the voice of others made in your workspace?	0.794
		Were you annoyed with the footsteps of others made in your ward?		Are you annoyed with the footsteps of others made in your workspace?	
		Were you annoyed with the machinery sounds made in your ward?		Are you annoyed with the machinery sounds made in your workspace?	
		Were you annoyed with the HVAC sounds made in your ward?		Are you annoyed with the HVAC sounds made in your workspace?	
		Were you annoyed with the sounds from the bathroom in your ward?		Are you annoyed with the sounds from the bathroom in your workspace?	
	Other space	Were you annoyed with the voice of others made in other ward?	0.950	N/A	
		Were you annoyed with the footsteps of others made in other ward?			
		Were you annoyed with the machinery sounds made in other ward?			
	Outdoors	Were you annoyed with the traffic sounds heard from outdoors?	0.948	Are you annoyed with the traffic sounds heard from outdoors?	0.859
		Were you annoyed with the sounds made by other outdoor activities?		Are you annoyed with the sounds made by other outdoor activities?	
Perception of helpfulness for recovery from COVID-19 5-point scale (1 “Not helpful at all” ∼ 5 “Totally helpful”)	Perceived help of the environments	In general, how much do you think the indoor environment helped you to recover from COVID-19?		In general, how much do you think the indoor environment helps your work at the COVID-19 ward?	

The study also aimed to explore the impacts of annoyance resulting from various noise events on respondents’ satisfaction with acoustic environments. In order to measure annoyance, the study employed a series of question items that specifically inquired about noise events originating from the same space, other space, and outdoors. Furthermore, the survey included a singular question measuring respondents’ perception of the helpfulness of either recovery from COVID-19 (for the patient group) or work (for the staff group).

To account for differences between respondent groups, the survey questions for the patient group were phrased in the past tense, as the respondents had been discharged at the time of the survey. In contrast, the questions posed to the staff group were phrased in the present tense, as the respondents were still working at their hospitals at the time of the survey. All questions were presented using a 5-point scale.

### 2.3. Procedure

**FIGURE 2:**
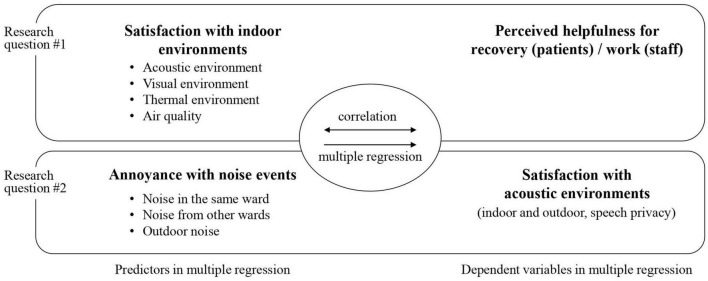
Data analysis procedure for investigating the relationships between the variables.

The online questionnaire survey was conducted during October and November of 2022. Respondents were recruited through a link posted online and distributed via email. Only individuals who were 19 years of age or older, and Republic of Korean citizens were eligible to participate. The participants provided their written informed consent to participate in this study. A total of 216 responses were initially collected. After screening, 13 responses were excluded, resulting in a final dataset of 203 responses (100 responses for the patient group and 103 responses for the staff group) that were used for the data analysis. Prior to participating in the survey, informed consent was obtained from the respondents. In order to ensure comparability between the data analyzed for the two groups, the number of responses was controlled to be similar. This was done to maintain balance and facilitate meaningful comparisons between the two groups. The relationships among the variables were explored through correlation and linear regression analyses, which were conducted using the Jamovi 2.3 (Fox and Weisberg, 2020; R Core Team, 2021; The jamovi project, 2022). Figure 2 illustrates how the relationships between the factors were analyzed.

## 3. Results

**FIGURE 3:**
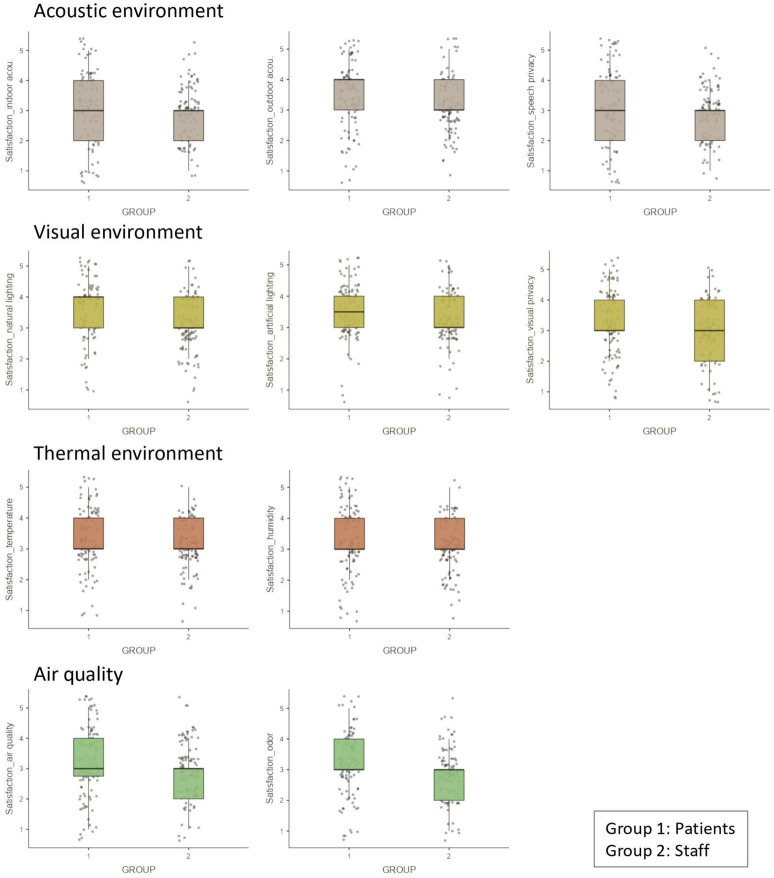
Boxplots illustrating the two groups’ satisfaction with indoor environments.

The Shapiro–Wilk test revealed that the data did not follow a normal distribution. Thus, Figure 3 presents how both groups reported satisfaction with hospital indoor environments in boxplots. It was found that satisfaction ratings of the patient group were generally higher compared to those of the staff group. Three tendencies were observed in the group differences. Firstly, both groups exhibited identical median values, but the patient group displayed higher 1st quartile values in some ratings including satisfaction with indoor acoustic environment and speech privacy. Secondly, the patient group exhibited higher median values in certain aspects, such as satisfaction with outdoor acoustic environment or satisfaction with natural and artificial lightings. Thirdly, the two groups demonstrated the same median values, but the staff group had lower 3rd quartile in some factors such as satisfaction with visual privacy. Among the tendencies highlighting the distinct patterns of satisfaction between the groups, it is noteworthy that satisfaction with thermal environment (temperature and humidity) exhibited a similar range of distributions. This suggests that both groups had comparable perceptions and experiences regarding the thermal conditions in the indoor environment.

Consequently, Spearman’s rank correlation coefficients were computed. Table 3 presents the correlation matrix between satisfaction with indoor environments and perceived helpfulness of the hospital environment. Table 3A displays the correlations for the patient group, while Table 3B presents the correlations for the staff group. All correlations between satisfaction with acoustic, visual, and thermal environments, as well as air quality, and perceived helpfulness were found to be positive and statistically significant. The associated *p*-values ranged from below 0.001 to 0.05, indicating a significant relationship between these variables.

**TABLE 3:** Correlation matrix between the satisfaction with indoor environments and perceived helpfulness of hospital environments.

		SAT_AC			SAT_VS			SAT_THM		SAT_AIR		HELP
		IN	OUT	PRV	NTR	ART	PRV	TMP	HMD	AIR	ODOR	
**(A) Patient group**												
Satisfaction with acoustic environment (SAT_AC)	Indoor (IN)	-										
	Outdoor (OUT)	0.549***	-									
	Speech privacy (PRV)	0.664***	0.427***	-								
Satisfaction with visual environment (SAT_VS)	Natural lighting (NTR)	0.556***	0.462***	0.521***	-							
	Artificial lighting (ART)	0.442***	0.547***	0.504***	0.662***	-						
	Visual privacy (PRV)	0.529***	0.448***	0.633***	0.575***	0.590***	-					
Satisfaction with thermal environment (SAT_THM)	Temperature (TMP)	0.508***	0.368***	0.625***	0.513***	0.575***	0.584***	-				
	Humidity (HMD)	0.522***	0.502***	0.568***	0.534***	0.664***	0.512***	0.813***	-			
Satisfaction with air quality (SAT_AIR)	Air quality (AIR)	0.479***	0.537***	0.555***	0.542***	0.621***	0.459***	0.634***	0.670***	-		
	Odor	0.438***	0.485***	0.510***	0.486***	0.588***	0.528***	0.565***	0.578***	0.733***	-	
Helpfulness of the environment for recovery from COVID-19 (HELP)		0.585***	0.522***	0.497***	0.393***	0.455***	0.416***	0.541***	0.523***	0.589***	0.608***	–
**(B) Staff group**												
Satisfaction with acoustic environment (SAT_AC)	Indoor (IN)	-										
	Outdoor (OUT)	0.435***	-									
	Speech privacy (PRV)	0.497***	0.451***	-								
Satisfaction with visual environment (SAT_VS)	Natural lighting (NTR)	0.251*	0.304**	0.444***	-							
	Artificial lighting (ART)	0.351***	0.431***	0.485***	0.547***	-						
	Visual privacy (PRV)	0.449***	0.287**	0.549***	0.506***	0.439***	-					
Satisfaction with thermal environment (SAT_THM)	Temperature (TMP)	0.354***	0.380***	0.492***	0.470***	0.388***	0.585***	-				
	Humidity (HMD)	0.349***	0.395***	0.406***	0.505***	0.470***	0.557***	0.698***	-			
Satisfaction with air quality (SAT_AIR)	Air quality (AIR)	0.412***	0.382***	0.474***	0.363***	0.355***	0.520***	0.551***	0.670***	-		
	Odor	0.344***	0.364***	0.375***	0.259**	0.262**	0.394***	0.482***	0.584***	0.690***	-	
Helpfulness of the environment for work at the COVID-19 ward (HELP)		0.385***	0.379***	0.316**	0.224*	0.320***	0.309**	0.361***	0.466***	0.462***	0.554***	–

*p < 0.05, **p < 0.01, and ***p < 0.001.

Additionally, Table 4 presents the correlation coefficients for satisfaction with acoustic environments and noise annoyance. The table shows negative correlations between the satisfaction and annoyance variables. In the patient group shown in Table 4A, annoyance with voice from other ward and outdoor noises (such as traffic and activities) did not exhibit significant correlations with satisfaction with indoor acoustic environment. Additionally, satisfaction with speech privacy in the patient group showed no correlation with any of the annoyance ratings pertaining to noise from other ward or outdoors. In Table 4B, which displays the correlations for the staff group, satisfaction with indoor acoustic environment did not correlate significantly with annoyance from sources such as voice, footsteps, machinery, bathroom noises heard within the same space, or outdoor noises. Similarly, satisfaction with outdoor acoustic environment did not demonstrate any significant correlations with footsteps, machinery, or HVAC noises heard within the same space. Furthermore, the staff group’s satisfaction with speech privacy did not correlate with annoyance from machinery noise within the same space.

**TABLE 4:** Correlation matrix between the satisfaction with acoustic environment and noise annoyance.

		SAT_AC			AN_SAME					AN_OTH			AN_OUT	
		IN	OUT	PRV	VC	FT	MCH	HVAC	BTH	VC	FT	MCH	TR	ACT
**(A) Patient group**														
Satisfaction with acoustic environment (SAT_AC)	Indoor (IN)	-												
	Outdoor (OUT)	0.549***												
	Speech privacy (PRV)	0.664***	0.427***											
Annoyance with noise in the same space (AN_SAME)	Voice (VC)	-0.457***	-0.316**	-0.468***										
	Foosteps (FT)	-0.315**	-0.269*	-0.305**	0.719***									
	Machinery (MCH)	-0.327***	-0.286**	-0.229*	0.545***	0.630***								
	HVAC	-0.467***	-0.337***	-0.305**	0.601***	0.598***	0.688***							
	Bathroom (BTH)	-0.277**	-0.399***	-0.259**	0.496***	0.676***	0.581***	0.582***						
Annoyance with noise in other space (AN_OTH)	Voice	-0.178	-0.216*	-0.173	0.312**	0.533***	0.498***	0.270**	0.505***					
	Foosteps (FT)	-0.206*	-0.282**	-0.151	0.378***	0.536***	0.547***	0.290**	0.568***	0.878***				
	Machinery (MCH)	-0.203*	-0.198*	-0.144	0.294**	0.445***	0.554***	0.285**	0.496***	0.831***	0.901***			
Annoyance with outdoor noise (AN_OUT)	Traffic (TR)	-0.148	-0.281**	-0.147	0.326**	0.401***	0.480***	0.300**	0.551***	0.677***	0.741***	0.722***		
	Activities (ACT)	-0.184	-0.246*	-0.145	0.360***	0.398***	0.499***	0.301**	0.567***	0.681***	0.758***	0.775***	0.908***	–
**(B) Staff group**														
Satisfaction with acoustic environment (SAT_AC)	Indoor (IN)	-												
	Outdoor (OUT)	0.435***	-											
	Speech privacy (PRV)	0.497***	0.451***	-										
Annoyance with noise in the same space (AN_SAME)	Voice (VC)	-0.112	-0.214*	-0.223*	-									
	Foosteps (FT)	-0.111	-0.169	-0.244*	0.650***	-								
	Machinery (MCH)	-0.069	-0.100	-0.156	0.491***	0.513***	-							
	HVAC	-0.248*	-0.064	-0.230*	0.284**	0.358***	0.459***	-						
	Bathroom (BTH)	-0.058	-0.335***	-0.261**	0.402***	0.610***	0.312**	0.390***	-					
Annoyance with outdoor noise (AN_OUT)	Traffic (TR)	-0.184	-0.341***	-0.231*	0.485***	0.550***	0.313**	0.321***	0.569***					
	Activities (ACT)	-0.182	-0.377***	-0.196*	0.407***	0.555***	0.275**	0.235*	0.604***				0.765***	–

*p < 0.05, **p < 0.01, and ***p < 0.001.

### 3.1. Patient group

To examine Research question 1-1, a multiple linear regression analysis was conducted using the data from the patient group. The objective was to predict the perceived helpfulness of recovery from COVID-19 based on satisfaction with various indoor environmental factors. The factors considered for satisfaction with indoor environments included three factors related to acoustic environment, three factors related to visual environment, two factors related to thermal environment, and two factors related to air quality. To assess multicollinearity, both tolerance and variance inflation factor (VIF) were calculated, and the results indicated an acceptable level of collinearity (Cohen et al., 2013). Table 5 shows all the regression coefficients for the tested predictors. The result showed that the regression model yielded an *R*-value of 0.768 (*R*^2^ = 0.589, Adjusted *R*^2^ = 0.536), which demonstrated a significant fit [*F*(10,77) = 11.0, *p* < 0.001, Durbin-Watson = 2.15]. It was revealed that satisfaction with indoor acoustic environment (β = 0.325, *p* < 0.01) and odor (β = 0.249, *p* < 0.05) were significant predictors of the perceived helpfulness of recovery from COVID-19.

**TABLE 5:** Regression coefficients for predictors of perceived helpfulness for recovery (patient group).

Predictor		*B*	β	*t*	*p*	*Fit*
**Dependent variable: perceived helpfulness for recovery**						
(Intercept)		0.523		1.376		*R*^2^ = 0.589 Adjusted *R*^2^ = 0.536 *p* < 0.001
Satisfaction with…	Indoor acoustic	0.304	0.325	2.853	**	
	Outdoor acoustic	0.201	0.199	1.915		
	Speech privacy	-0.099	-0.111	-0.901		
	Natural lighting	-0.063	-0.058	-0.513		
	Artificial lighting	-0.098	-0.080	-0.632		
	Visual privacy	-0.013	-0.013	-0.108		
	Temperature	0.213	0.195	1.257		
	Humidity	0.112	0.113	0.713		
	Air quality	0.096	0.101	0.761		
	Odor	0.262	0.249	2.075	*	

*p < 0.05 and **p < 0.01.

To address Research question 2 for the patient group, separate multiple linear regression models were employed. The purpose of these analyses was to predict patients’ satisfaction with acoustic environment using multiple variables of noise annoyance. These variables encompassed annoyance ratings associated with noise sources within the same ward, other ward, and outdoors (Table 6). Collinearity statistics were assessed, and one variable (i.e., annoyance with footstep noise from other space) was removed as it displayed a VIF of 10.2 and tolerance of 0.1. The remaining variables were within acceptable limits for tolerance and VIF. Table 6 shows all the regression coefficients for the tested predictors. Among the three models tested, two were found to be significant. First, satisfaction with indoor acoustic environment was predicted. The resulting model yielded an *R*-value of 0.598 (*R*^2^ = 0.357, Adjusted *R*^2^ = 0.296) with a significant model fit [*F*(7,73) = 5.80, *p* < 0.001, Durbin-Watson = 1.89]. The findings indicated that annoyance with other people’s voice in the same ward (β = −0.311, *p* < 0.05) and HVAC noise in the same ward (β = −0.441, *p* < 0.01) were significant predictors of satisfaction with indoor acoustic environment. Second, satisfaction with speech privacy was found to be predicted with annoyance with voice of others in the same ward (β = −0.553, *p* < 0.01) and from other ward [(β = −0.490, *p* < 0.05)]. The model presented *R* of 0.537 (*R*^2^ = 0.288, Adjusted *R*^2^ = 0.220) with a significant model fit [*F*(7,73) = 4.22, *p* < 0.001, Durbin–Watson = 1.94]. Lastly, satisfaction with outdoor acoustic environment was predicted based on outdoor noise annoyance, but the result showed that the model was not statistically significant.

**TABLE 6:** Regression coefficients for predictors of satisfaction with acoustic environment (patient group).

Predictor		*B*	β	*t*	*p*	*Fit*
**Dependent variable: satisfaction with indoor acoustic environment**						
(Intercept)		4.698		12.933	***	*R*^2^ = 0.357 Adjusted *R*^2^ = 0.296 *p* < 0.001
Noise annoyance due to…	Same ward_voice	-0.305	-0.311	-2.021	*	
	Same ward_footstep	0.120	0.113	0.629		
	Same ward_machinary	-0.121	-0.126	-0.811		
	Same ward_HVAC	-0.432	-0.441	-2.962	**	
	Same ward_bathroom	0.278	0.265	1.839		
	Other ward_voice	-0.362	-0.384	-1.679		
	Other ward_machine	0.298	0.308	1.363		
**Dependent variable: satisfaction with speech privacy**						
(Intercept)		4.556		11.820	***	*R*^2^ = 0.288 Adjusted *R*^2^ = 0.220 *p* < 0.001
Noise annoyance due to…	Same ward_voice	-0.546	-0.553	-3.417	**	
	Same ward_footstep	0.231	0.216	1.143		
	Same ward_machine	-0.130	-0.135	-0.823		
	Same ward_HVAC	-0.033	-0.034	-0.216		
	Same ward_bathroom	0.026	0.025	0.162		
	Other ward_voice	-0.465	-0.490	-2.036	*	
	Other ward_machine	0.441	0.452	1.901		

*p < 0.05, **p < 0.01, and ***p < 0.001.

The model predicted satisfaction with outdoor acoustic environment was not statistically significant.

### 3.2. Staff group

To investigate Research question 1-2, the staff group’s perceived helpfulness for work was predicted using satisfaction with indoor environments as predictors. The tested predictors consisted of three factors related to acoustic environment, three factors related to visual environment, two factors related to thermal environment, and two factors related to air quality. Table 7 presents the regression coefficients for all the tested predictors in the model. It also presents the results showing the acceptable level of collinearity and the model presented *R* of 0.641 (*R*^2^ = 0.411) with a significant fit [*F*(10,92) = 6.41, *p* < 0.001, Durbin–Watson = 1.84]. It was found that only satisfaction with odor (β = 0.440, *p* < 0.001) significantly predict the perceived helpfulness for work at the COVID-19 ward.

**TABLE 7:** Regression coefficients for predictors of perceived helpfulness for work (staff group).

Predictor		*B*	β	*t*	*p*	*Fit*
**Dependent variable: perceived helpfulness for work**						
(Intercept)		0.918		2.532	*	*R*^2^ = 0.411 Adjusted *R*^2^ = 0.347 *p* < 0.001
Satisfaction with…	Indoor acoustic	0.173	0.174	1.620		
	Outdoor acoustic	0.094	0.097	0.933		
	Speech privacy	0.025	0.025	0.213		
	Natural lighting	-0.049	-0.050	-0.432		
	Artificial lighting	0.091	0.085	0.741		
	Visual privacy	-0.037	-0.040	-0.326		
	Temperature	-0.055	-0.048	-0.351		
	Humidity	0.140	0.125	0.826		
	Air quality	-0.028	-0.029	-0.217		
	Odor	0.430	0.440	3.639	***	

*p < 0.05 and ***p < 0.001.

To examine Research question 2 for the staff group, a prediction model was constructed to assess satisfaction with acoustic environment using noise annoyance as predictors. The findings, presented in Table 8, indicated an acceptable level of collinearity within the model. However, the tested model for predicting satisfaction ratings with the indoor acoustic environment did not reach statistical significance. While it is important to distinguish between correlation and causal relationships, the correlation coefficients presented in Table 4B can provide insight into the observed results. The lack of significance in the regression model aligns with the limited correlations found earlier. The correlation analysis revealed few associations between the tested variables, with only HVAC noise annoyance exhibiting a significant but weak correlation with the satisfaction factor. These findings suggest that the variables may not possess a strong linear relationship or direct causal influence on each other. Furthermore, the results indicated that there was no significant predictor of satisfaction with speech privacy. The lack of significance in the predictors can also be attributed to the weak correlations observed between the tested variables. The limited associations found in the correlation analysis may contribute to the absence of strong predictors for satisfaction with speech privacy. Only satisfaction with outdoor acoustic environment was significantly predicted by annoyance with outdoor traffic noise (β = −0.336, *p* < 0.05). The model presented *R*-value of 0.403 (*R*^2^ = 0.162) with a significant model fit [*F*(2,100) = 9.68, *p* < 0.001, Durbin–Watson = 1.91].

**TABLE 8:** Regression coefficients for predictors of satisfaction with acoustic environment (staff group).

Predictor		*B*	β	*t*	*p*	*Fit*
**Dependent variable: satisfaction with outdoor acoustic environment**						
(Intercept)		4.130		17.140	***	*R*^2^ = 0.162 Adjusted *R*^2^ = 0.145 *p* < 0.001
Noise annoyance due to…	Outdoors_activities	-0.079	-0.083	-0.597		
	Outdoors_traffic	-0.301	-0.336	-2.408	*	

*p < 0.05 and ***p < 0.001.

The model predicted satisfaction with indoor acoustic environment was not statistically significant.

In the model predicting satisfaction with speech privacy, none of the predictors were significant.

## 4. Discussion

### 4.1. General discussion

The purpose of this study was to examine relationships between factors regarding indoor environment of COVID-19 hospitals. The first objective was to investigate how the indoor environment of the hospital affects the perception of patients and staff. Patients were asked to evaluate how helpful the hospital environment was in aiding their recovery from COVID-19, while staff members were asked to assess the helpfulness of the environment in the COVID-19 ward for their work. The second objective focused on the relationship between annoyance with different noise events in the hospital environment and satisfaction with acoustic environment, as perceived by the occupants.

The study’s results demonstrated the crucial importance of indoor environmental elements in patients’ perception of how beneficial their hospital experience was for their recovery. These findings have significant implications for healthcare providers that they should prioritize the quality management of indoor environmental factors, such as sound and air quality, as part of their overall patient care strategy. Addressing these factors may help reduce patients’ stress levels, promote relaxation, and improve their overall hospital experience (Walker and Karl, 2019; Zhou et al., 2020; Pantaleon and Lewis, 2022). Moreover, ensuring healthy indoor environments can also reduce the risk of other health complications so that it can lead to better patient outcomes and shorter hospital stays.

Satisfaction with indoor acoustic environment was one of the significant factors impacting the patient group’s perception of recovery. Furthermore, the results revealed that this particular factor, satisfaction with indoor acoustic environment, was influenced by annoyance with voice and HVAC noise heard within the same ward. It is essential to control and address this factor of annoyance to enhance the overall acoustic environment. Since the participants of the patient group were in COVID-19 ward, it’s possible that the HVAC systems were operating at full capacity, which could have affected the patients’ annoyance with HVAC noise. Improving indoor acoustic insulation performance is recommended in order to mitigate noise-related issues. Previous research has suggested that implementing better acoustic materials and optimizing space layout can improve patient health and enhance the overall quality of the indoor environment in healthcare facilities (Hagerman et al., 2005; Dascalaki et al., 2009). Besides, voice from either the same or other ward was identified as significant predictors affecting patients’ satisfaction with speech privacy. Voice has been known to be one of the major noise sources in hospital settings, impacting patients’ rest and sleep and reducing occupants’ speech privacy (Basner et al., 2014; Jue and Nathan-Roberts, 2019). To enhance patients’ hospital experiences and promote faster recovery, designers of healthcare facilities should consider reducing noise exposure and improving soundscapes. For example, maximum number of beds in one ward can be reduced to control exposure to others’ voices, or sound-absorbing materials can be installed on ceilings and walls to improve the acoustic quality (Harris, 2015; Farrehi et al., 2016; Jiang and Verderber, 2017).

Odor was one of two significant predictors for the patient group’s recovery perception and the only predictor for the staff group’s perceived helpfulness for work. Although all the occupants needed to wear facial masks indoors, odor was found to be a significant factor affecting the occupants’ perception. This finding suggests that while patients and staff both have unique and important perspectives on the indoor environment of hospitals, their priorities and concerns may differ. Patients may prefer an acoustic environment that is conducive to rest and recovery. Conversely, for medical staff, managing and eliminating unpleasant odors may be a priority in promoting a positive and productive work environment (Salamone et al., 2021; Ortiz and Bluyssen, 2022). This may involve implementing odor control measures, such as regular cleaning and disinfecting, proper waste disposal, and ensuring adequate ventilation systems (Horiguchi et al., 2015).

It is possible that the strong odors of chemicals and medicine present in the hospital environment could have influenced the participants’ perception. Thus, it is important for healthcare providers to prioritize the management of indoor air quality, including the control of strong odors, as part of their overall strategy to ensure a safe and healthy environment for both patients and staff.

Besides, the staff group did not show any significant impact of perception of acoustic environment on their perception of helpfulness for work. Only annoyance with traffic noise was a significant predictor for satisfaction with outdoor acoustic environment. However, the lack of significance for the staff group’s perception of the acoustic environment on their perceived helpfulness for work may not necessarily mean that noise and sound quality are not important factors. It is possible that staff members may have already acclimated to the noise level in their workplace and therefore do not perceive it as significantly impacting their work performance. It is also important to consider that different job characteristics can influence how the acoustic environment affects employees’ work (Park et al., 2020). In the case of healthcare professionals, such as the staff group involved in the present study, their job roles may involve frequent communication. As a result, factors related to the acoustic environment may not have a significant impact on their work performance. Nonetheless, efforts should still be made to recognize the potential negative effects of high noise levels, including stress and fatigue, and to create a more conducive and supportive work environment for staff members (Parthasarathy and Tobin, 2004; Basner et al., 2014; Awada et al., 2021).

### 4.2. Limitation and future study recommendations

The present study has limitations that can be considered in future research. First, it did not collect objective data on each IEQ attribute, which could have provided a more detailed understanding of the factors affecting occupants’ experiences. Since the study solely relied on self-reported data, it might have been subject to recall bias. However, the study did yield valuable insights into general subjective responses to indoor environments of COVID-19 hospitals. Future research could build on this by targeting specific sites and measuring each environmental quality in detail, while also capturing occupants’ subjective assessments of those qualities. By combining objective and subjective data in this way, future research could gain a more understanding of the relationship between IEQ and occupants’ experiences in healthcare settings. Moreover, incorporating in-depth interviews with occupants could also provide valuable insights into the topic. While quantitative studies such as this one can shed light on tendency in the collected data, there may still be unknown issues that can only be uncovered through qualitative research methods. Therefore, combining quantitative and qualitative approaches can lead to a more comprehensive understanding of the factors that influence occupants’ experiences in healthcare settings.

The study aimed to address two major research questions. It could be examined simultaneously through the use of structural equation modeling. However, this method requires a larger sample size than that of the present study. While it may have been possible to collect additional responses from patients, the same was not true for the staff group because it was more challenging to recruit participants of the staff group. Therefore, to ensure a balanced representation of both patient and staff perspectives, the present study collected a similar number of responses from each group and employed multiple regression models to analyze the data. Although multiple regression models did not allow for simultaneous examination of several relationships between variables, it still provided valuable insights into the perceptions of patients and staff regarding indoor environment in COVID-19 hospitals. Future studies with larger sample sizes could consider the use of structural equation modeling to investigate the associations between the variables more comprehensively.

The present study primarily focused on investigating the acoustic environment and its focus was on noise annoyance. However, it is important to acknowledge that assessing annoyance might have caused a negative response bias among the participants. Future research can employ alternative methods such as exploring the perception of soundscape using the ISO 12913 series to assess occupants’ affective reactions to the acoustic environment, and its impacts on their health and wellbeing. To fully understand how occupants perceive the acoustic environment, it is important to consider its interactions with other environmental factors more in-depth. For example, future research could investigate the influence of natural and artificial lightings on acoustic perception. By taking a more comprehensive approach to the study of indoor environmental qualities, researchers can gain valuable insights into how to manage hospital environments holistically, leading to better patient outcomes and a higher quality of care. Also, further research can contribute to the development of evidence-based guidelines and standards for the acoustic performance of hospital environments. By establishing such guidelines, designers and healthcare providers can ensure that hospital environments provide a better space with occupant comfort for both patients and staff, ultimately improving the overall healthcare and workplace experience.

## 5. Conclusion

The present study offered valuable insights into the perceptions of patients and staff regarding indoor environments in COVID-19 hospitals in Republic of Korea. Through data collection and analysis, the study explored the relationships between various variables. The results showed that the patient groups’ satisfaction with indoor acoustic environment and odor significantly affected their perceived helpfulness for recovery. Similarly, satisfaction with odor was a significant predictor for the staff group’s perceived helpfulness for work. Furthermore, annoyance caused by voice and HVAC noise in the same ward significantly influenced the patient group’s satisfaction with indoor acoustic environment. In addition, annoyance due to voice heard from the same or other ward had a significant impact on the patient group’s satisfaction with speech privacy. In contrast, no significant relationship was found between satisfaction with indoor acoustic environment and indoor noise annoyance in the staff group. Only annoyance with outdoor traffic noise served as a significant predictor for their satisfaction with outdoor acoustic environment. Overall, the findings in the present study underscored the importance of considering the perspectives of both patients and staff in the design and management of hospital environments.

## Data availability statement

The raw data supporting the conclusions of this article will be made available by the authors, without undue reservation.

## Ethics statement

Ethical review and approval was not required for the study on human participants in accordance with the local legislation and institutional requirements. The patients/participants provided their written informed consent to participate in this study.

## Author contributions

SP, HS, and KK contributed to the conception and design of the study as well as development of the survey questionnaire. SP collected and analyzed the data, and wrote the first draft of the manuscript. All authors contributed to the manuscript revision and approved the submitted version.
